# A Low-Cost Electrochemical Cell Sensor Based on MWCNT-COOH/α-Fe_2_O_3_ for Toxicity Detection of Drinking Water Disinfection Byproducts

**DOI:** 10.3390/nano15020146

**Published:** 2025-01-20

**Authors:** Ying Liu, Zhipeng Zhang, Yuling Wu, Huan Yang, Jiao Qu, Xiaolin Zhu

**Affiliations:** School of Environment, Northeast Normal University, Changchun 130117, China

**Keywords:** biosensor, disinfection byproducts, cytotoxicity, multi-walled carbon nanotubes, nano-iron (III) oxide

## Abstract

The disinfection of drinking water is essential for eliminating pathogens and preventing waterborne diseases. However, this process generates various disinfection byproducts (DBPs), which toxicological research indicates can have detrimental effects on living organisms. Moreover, the safety of these DBPs has not been sufficiently assessed, underscoring the need for a comprehensive evaluation of their toxic effects and associated health risks. Compared to traditional methods for studying the toxicity of pollutants, emerging electrochemical sensing technologies offer advantages such as simplicity, speed, and sensitivity, presenting an effective means for toxicity research on pollutants. However, challenges remain in this field, including the need to improve electrode sensitivity and reduce electrode costs. In this study, a pencil graphite electrode (PGE) was modified with carboxylated multi-walled carbon nanotubes (MWCNT-COOH) and nano-iron (III) oxide (α-Fe_2_O_3_) to fabricate a low-cost electrode with excellent electrocatalytic performance for cell-active substances. Subsequently, a novel cellular electrochemical sensor was constructed for the sensitive detection of the toxicity of three drinking water DBPs. The half inhibitory concentration (IC_50_) values of 2-chlorophenylacetonitrile (2-CPAN), 3-chlorophenylacetonitrile (3-CPAN), and 4-chlorophenylacetonitrile (4-CPAN) for HepG2 cells were 660.69, 831.76, and 812.83 µM, respectively. This study provides technical support and scientific evidence for the toxicity detection and safety assessment of emerging contaminants.

## 1. Introduction

Drinking water disinfection is considered one of the greatest public health achievements of the 20th century, as it is vital for eliminating pathogenic microorganisms and preventing the spread of waterborne diseases. However, during disinfection processes, disinfectants react with natural organic matter, anthropogenic pollutants, and inorganic ions present in source water, resulting in the formation of numerous disinfection by-products (DBPs) [1,2]. Since the identification of trihalomethanes (THMs) in 1974, over 700 DBPs have been detected [3,4]. In recent years, a significant increase in the detection of aromatic disinfection by-products in drinking water has been observed. Toxicological studies indicate that these aromatic DBPs generally possess higher levels of cytotoxicity, genotoxicity, developmental toxicity, and growth inhibition when compared to aliphatic DBPs [5]. Chlorochloroacetonitrile is a highly stable aromatic nitrogen-containing compound formed during chlorination and chloramine disinfection processes. It has been measured in drinking water at concentrations in the ng/L range [6]. Toxicological assessments of 2-chlorophenylacetonitrile (2-CPAN) using Chinese hamster ovary cells have revealed significant toxicity, with 2-CPAN exhibiting toxicity that is 61 times greater than that of regulated trichloromethane [7]. Furthermore, 3-chlorophenylacetonitrile (3-CPAN) and 4-chlorophenylacetonitrile (4-CPAN) have recently been detected in drinking water [8]. Consequently, there is an urgent need for rapid and accurate evaluations of the toxic effects of these DBPs in drinking water.

Traditional methods for assessing the cytotoxicity of environmental pollutants, such as the MTT assay, lactate dehydrogenase (LDH) assay, and flow cytometry, have several limitations, including high costs, complex procedures, and the necessity for staining [9,10]. Therefore, there is a pressing need to develop a rapid, sensitive, cost-effective, non-toxic, and staining-free evaluation method. Recently, an electrochemical approach based on nucleotide metabolism has emerged as a novel technique for detecting the cytotoxicity of pollutants [11]. This method assesses cell viability by measuring the electrochemical signals of nucleotide metabolites, such as guanine and xanthine, which reflect the toxic effects of pollutants on cells [12]. It offers several advantages, including simplicity, speed, non-toxicity, and the absence of labeling [13], thereby showing promise for applications in environmental toxicology research [14]. However, there are currently several issues in this field, including the high cost of electrodes, the need for improved electrode sensitivity, and the relatively complex surface treatment and modification of electrodes. In comparison to commonly used glassy carbon electrodes, lead core electrodes are significantly less expensive, costing approximately one-thousandth as much. Additionally, they offer a wide electrochemical window, ease of modification, and the ability to reduce surface passivation effects [15,16]. To improve the sensitivity of electrochemical detection, it is essential to modify the electrodes with high-performance novel nanomaterials.

Multi-walled carbon nanotubes (MWCNTs) are characterized by a large specific surface area and excellent conductivity, making them widely utilized in the fabrication of electrochemical sensors. Functionalization treatments, such as hydroxylation or carboxylation, improve their dispersibility, increase the electroactive surface area, and enhance electrocatalytic performance [17,18,19]. However, the electroactive surface area of electrochemical sensors prepared solely using functionalized MWCNTs modified electrodes remains limited, and their catalytic performance is insufficient for the sensitive detection of electrochemical signals from cells. Therefore, composite materials combining MWCNTs with other nanomaterials are necessary. Nano-iron (III) oxide (α-Fe_2_O_3_) is a transition metal oxide nanomaterial that exhibits high specific surface area, good chemical stability, and biocompatibility compared to conventional iron materials [20,21]. The combination of MWCNTs and α-Fe_2_O_3_ is anticipated to increase the number of active reaction sites, thereby enhancing electrocatalytic performance and electron transfer efficiency. Conventional glassy carbon electrodes based on MWCNTs and α-Fe_2_O_3_ have been successfully used for the electrochemical detection of certain environmental pollutants and drugs, such as benzoquinone, catechol, and ivabradine [22,23].

In this study, the cost-effective pencil graphite electrode (PGE) with a diameter of 2 mm was chosen as the substrate. Carboxylated multi-walled carbon nanotubes (MWCNT-COOH)/α-Fe_2_O_3_ composite materials were synthesized using a simple green methodology, and MWCNT-COOH/α-Fe_2_O_3_/PGE materials were prepared via a drop-coating technique. A cell-based electrochemical sensor was constructed using human hepatocellular carcinoma (HepG2) cells as a model. The MWCNT-COOH/α-Fe_2_O_3_ composite materials were characterized using scanning electron microscopy (SEM), energy-dispersive X-ray spectroscopy (EDS), X-ray diffraction (XRD), Fourier transform infrared spectroscopy (FTIR), and cyclic voltammetry (CV). Additionally, the cytotoxic effects of 2-CPAN, 3-CPAN, and 4-CPAN were investigated.

## 2. Materials and Methods

### 2.1. Instruments and Reagents

2-CPAN (99%), 3-CPAN (≥98%), and 4-CPAN (≥98%) were purchased from J&K Scientific Ltd., Beijing, China. MWCNT-COOH (purity: 95%, diameter: 30–50 nm, length: 0.5–2 µm) was obtained from Jiangsu XFNANO Materials Tech Co., Ltd., Nanjing, China. α-Fe_2_O_3_ (purity: 99.5%, particle size: 30 nm) was purchased from Aladdin Reagents Co., Ltd., Shanghai, China. PGE (type: 2 B, diameter: 2 mm) was obtained from Shanghai M&G Stationery Inc., Shanghai, China. Fetal bovine serum (FBS), DMEM culture medium, penicillin, streptomycin, serum, and phosphate-buffered saline (PBS) were all purchased from Corning, Corning, NY, USA. HepG2 cells were obtained from the Cell Bank/Stem Cell Bank of the Chinese Academy of Sciences. The cells were cultured at 37 °C in a 5% CO_2_ incubator in a medium containing 10% fetal bovine serum and 1% antibiotics (penicillin and streptomycin).

The morphology and structure of the MWCNT-COOH/α-Fe_2_O_3_ composite material were characterized using SEM (Zeiss Gemini Sigma 300, Jena, Germany), EDS (Ultim Max 100, London, UK), XRD (Rigaku Dmax Ultima+, Tokyo, Japan), and FTIR (Thermo Fisher Scientific Nicolet iS20, Waltham, MA, USA). All electrochemical experiments were conducted using a CHI630e electrochemical workstation. Cell morphology was observed using an inverted biological microscope (BDS-400, Chongqing, China).

### 2.2. Preparation of MWCNT-COOH/α-Fe_2_O_3_/PGE

The PGE was polished to a mirror finish using Al_2_O_3_ powder on a polishing cloth, followed by thorough cleaning with double-distilled water and air drying. A 5 µL drop of MWCNT-COOH aqueous dispersion containing 80% (*v*/*v*) N,N-dimethylformamide (DMF) was applied to the surface of the PGE and dried under an infrared lamp to obtain MWCNT-COOH/PGE. A suitable amount of α-Fe_2_O_3_ powder was then added to the 0.8 mg/mL MWCNT-COOH aqueous dispersion and subjected to ultrasonic treatment for 30 min. A 5 µL drop of the MWCNT-COOH/α-Fe_2_O_3_ dispersion was subsequently applied to the polished surface of a PGE and dried under an infrared lamp to yield MWCNT-COOH/α-Fe_2_O_3_/PGE.

### 2.3. Construction and Condition Optimization of Electrochemical Sensor

The three-electrode system is composed of MWCNT-COOH/α-Fe_2_O_3_/PGE as the working electrode, a saturated calomel electrode as the reference electrode, and a platinum electrode as the counter electrode. Using chronoamperometry, an enrichment time of 300 s was set, and the enrichment potentials were as follows: −0.3 V, −0.1 V, 0 V, +0.1 V, and +0.3 V. The influence of enrichment potentials on the peak current of HepG2 cells was investigated to establish the optimal enrichment conditions. The same electrode was used to continuously measure the same concentration of HepG2 cell samples ten times, and the relative standard deviation (RSD) of the oxidation peak current was calculated to evaluate the repeatability of the electrochemical sensor. Based on the optimized detection conditions, an electrochemical sensor based on MWCNT-COOH/α-Fe_2_O_3_/PGE was constructed. Prior to its first use, the prepared MWCNT-COOH/α-Fe_2_O_3_/PGE was scanned for 15 cycles in a 0.2 M PBS using cyclic voltammetry (CV) within a voltage range of 0 to +0.8 V.

### 2.4. Cytotoxicity Detection

Cells were cultured in an environment maintained at 37 °C and 5% CO_2_. To support the growth and proliferation of HepG2 cells, DMEM complete medium supplemented with 10% fetal bovine serum was utilized. To ensure cell viability and optimal growth conditions, passaging was conducted every two days. Prior to the toxicity testing, HepG2 cells were allowed a 6 h period to grow and adhere, ensuring their adaptation to the new culture environment and enabling stable proliferation. To evaluate the toxic effects of different concentrations of drinking water disinfection by-products on HepG2 cells, three sets of parallel experiments were established. The control group consisted of untreated culture medium, while the experimental groups were exposed to different concentrations of drinking water disinfection by-products: 2-CPAN (125.89 µM, 251.19 µM, 501.19 µM, 1000.00 µM, 1995.26 µM), 3-CPAN (125.89 µM, 251.19 µM, 501.19 µM, 1000.00 µM, 1995.26 µM), and 4-CPAN (125.89 µM, 251.19 µM, 501.19 µM, 1000.00 µM, 1995.26 µM).

After 36 h of cell cultivation, the culture dishes were removed for subsequent cell lysis and electrochemical detection. After PBS (pH 7.4) was added to the culture dishes, they were heated in a 50 °C constant temperature water bath for 0.5 h to promote the lysis of HepG2 cells, thereby preparing the HepG2 cells lysate. The electrochemical signals of the HepG2 cells lysate were detected using MWCNT-COOH/α-Fe_2_O_3_/PGE. The cytotoxicity was calculated using the following Formula (1) [24]:

(1) $$Cytotoxicity=\left[\left(I_{pa,1}-I_{pa,2}\right)/I_{pa,1}\right]\times 100\%$$

where *I_pa_*_,1_ and *I_pa_*_,2_ represent the peak current values for the control group and the experimental group cells, respectively. The half inhibitory concentration (IC_50_) was calculated using this formula to compare the toxic effects.

## 3. Results and Discussion

### 3.1. Characterization of MWCNT-COOH/α-Fe_2_O_3_ Composite

**Figure 1:**
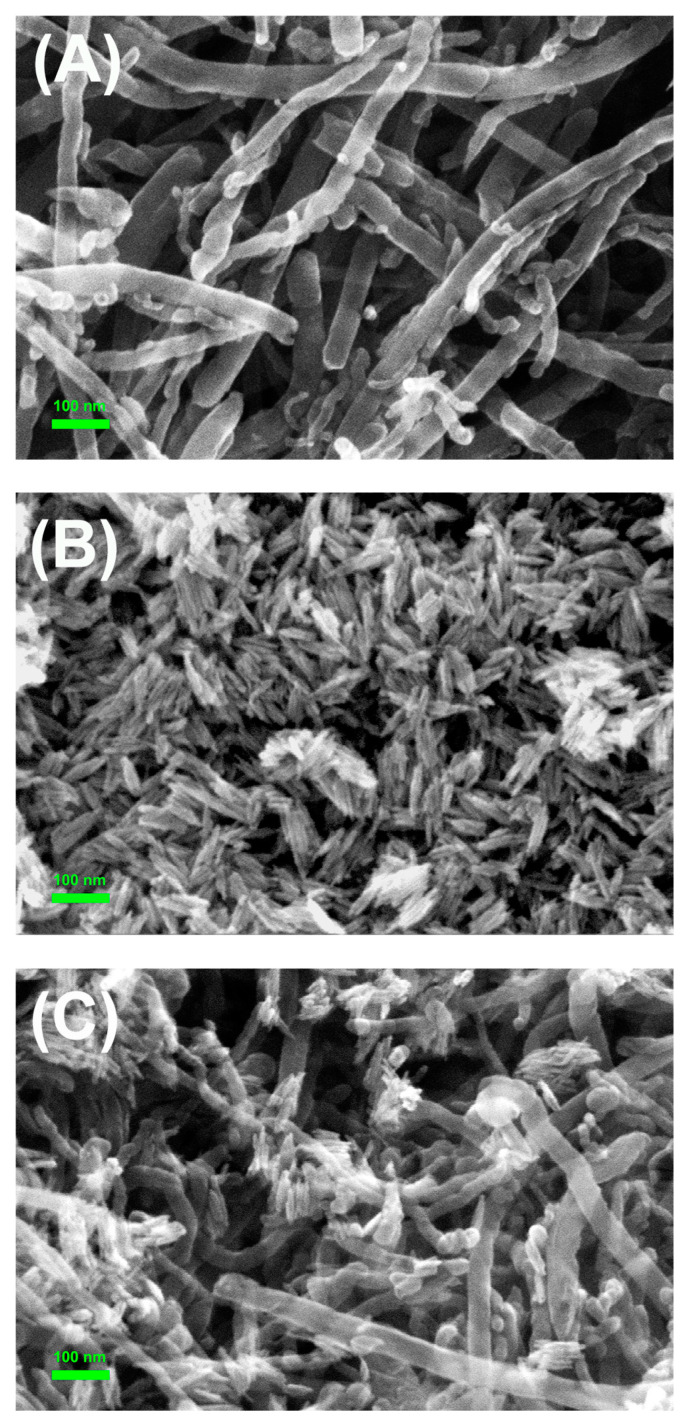
SEM images of α-Fe_2_O_3_ (**A**), MWCNT-COOH (**B**), and MWCNT-COOH/α-Fe_2_O_3_ (**C**).

The morphology of α-Fe_2_O_3_, MWCNT-COOH, and MWCNT-COOH/α-Fe_2_O_3_ was characterized using SEM (Figure 1). α-Fe_2_O_3_ exhibited a rod-like structure with a particle size of less than 30 nm (Figure 1A), while MWCNT-COOH had an intertwined tubular structure (Figure 1B) [25,26]. After the two materials were combined, α-Fe_2_O_3_ can be clearly observed to be loaded on the surface of MWCNT-COOH, indicating the successful preparation of the MWCNT-COOH/α-Fe_2_O_3_ binary nanocomposite (Figure 1C).

**Figure 2:**
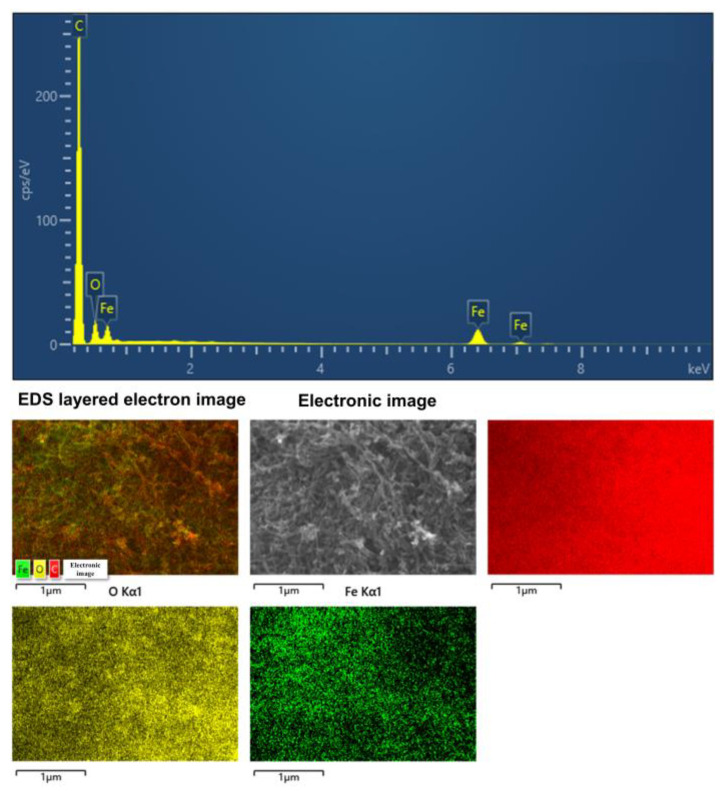
EDS spectrum of MWCNT-COOH/α-Fe_2_O_3_.

The elemental composition of the MWCNT-COOH/α-Fe_2_O_3_ composite was analyzed using EDS (Figure 2). Peaks attributed to C, O, and Fe elements were detected at 0.25 keV, 0.58 keV, and 0.72 keV in the EDS spectrum. The layered EDS mapping images indicate a uniform distribution of C, O, and Fe, confirming the successful preparation of the MWCNT-COOH/α-Fe_2_O_3_ composite.

**Figure 3:**
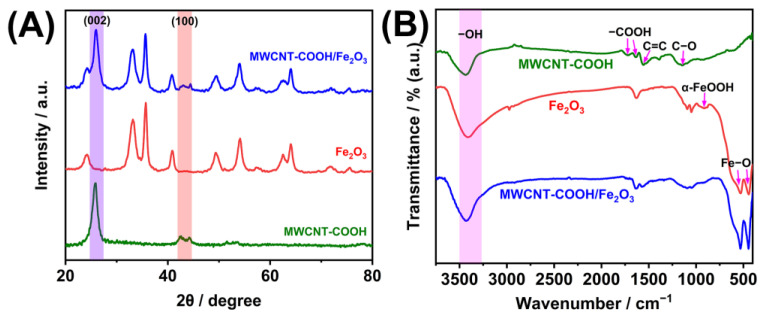
XRD (**A**) and FTIR spectra (**B**) of MWCNT-COOH, α-Fe_2_O_3_, and MWCNT-COOH/α-Fe_2_O_3_.

The crystal structures of MWCNT-COOH, α-Fe_2_O_3_, and MWCNT-COOH/α-Fe_2_O_3_ were characterized using XRD (Figure 3A). In the spectrum of MWCNT-COOH, two peaks appeared at 2θ = 25.82° and 2θ = 42.59°, corresponding to the (002) crystal plane of the graphite-like structure and the (100) crystal plane of the periodic structure of the carbon nanotube graphene layer, respectively [27]. The α-Fe_2_O_3_ spectrum reveals several sharp characteristic peaks attributed to α-Fe_2_O_3_, indicating a relatively high degree of crystallinity [28]. In the MWCNT-COOH/α-Fe_2_O_3_ spectrum, the characteristic diffraction peaks of both MWCNT-COOH and α-Fe_2_O_3_ are present and show no significant shifts, confirming the successful formation of the composite material.

FTIR analysis was conducted to examine the surface functional groups of MWCNT-COOH, α-Fe_2_O_3_, and the MWCNT-COOH/α-Fe_2_O_3_ composite (Figure 3B). The FTIR spectrum of MWCNT-COOH reveals absorption peaks at 3432, 1725, 1641, and 1068 cm^−1^, corresponding to the functional groups -OH, -COOH, C = C, and C-O, respectively [29,30]. In the spectrum of α-Fe_2_O_3_, characteristic vibration peaks for Fe-O were observed at 440 and 530 cm^−1^, along with an absorption peak for α-FeOOH at 915 cm^−1^; these findings confirm the rhombohedral crystal structure of the α-Fe_2_O_3_, specifically identifying it as α-Fe_2_O_3_ [31,32]. The spectrum of MWCNT-COOH/α-Fe_2_O_3_ exhibited the characteristic absorption peaks of both MWCNT-COOH and α-Fe_2_O_3_ without the emergence of any new peaks, indicating the successful synthesis of the MWCNT-COOH/α-Fe_2_O_3_ nanocomposite.

### 3.2. Optimization of Preparation Conditions for MWCNT-COOH/α-Fe_2_O_3_/PGE

When preparing modified electrodes using the drop-coating method, the quantity of modifying material plays a crucial role in determining the performance of the electrochemical sensor. In previous studies [13], it has been confirmed that the electrochemical signals generated by HepG2 cells in the 0–0.8 V range are attributable to xanthine and guanine. Therefore, the impact of various concentrations (0.2, 0.4, 0.6, 0.8, and 1.0 mg/mL) of 5 µL of MWCNT-COOH on the oxidation peak current of xanthine and guanine was investigated (Figure S1A). The findings indicated that the oxidation peak current for xanthine and guanine was maximized at a concentration of 0.8 mg/mL of MWCNT-COOH in the prepared MWCNT-COOH/PGE electrode. Consequently, we selected 0.8 mg/mL as the optimal concentration for subsequent modifications of the materials.

Additionally, the ratio of composite materials significantly influences the performance of the modified electrode. Under the optimal preparation conditions for MWCNT-COOH/PGE, we explored the ideal composite ratio of α-Fe_2_O_3_ to MWCNT-COOH by varying their mass ratios (0.125, 0.5, 1.0, 1.5, 2.0). The results demonstrated that the maximum purine oxidation peak current occurred at a mass ratio of 1.5 (α-Fe_2_O_3_ to MWCNT-COOH) (Figure S1B), indicating optimal performance of the MWCNT-COOH/α-Fe_2_O_3_/PGE composite. Therefore, a mass ratio of 1.5 for α-Fe_2_O_3_ to MWCNT-COOH was determined to be the optimal condition for synthesizing MWCNT-COOH/α-Fe_2_O_3_/PGE.

### 3.3. Construction of Cell Electrochemical Sensor Based on MWCNT-COOH/α-Fe_2_O_3_/PGE

**Figure 4:**
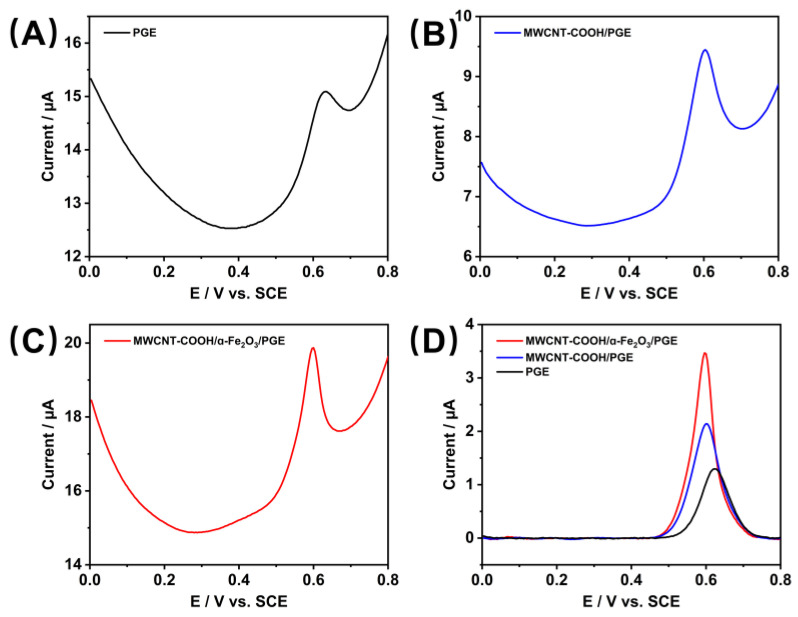
Differential pulse voltammetry of HepG2 cell lysate on (**A**) PGE, (**B**) MWCNT-COOH/PGE, and (**C**) MWCNT-COOH/α-Fe_2_O_3_/PGE and (**D**) baseline corrected differential pulse voltammetry of different electrodes in HepG2 cell lysate (Initial potential: 0 V; final potential: 0.8 V; voltage increment: 0.004 V; amplitude: 0.05 V pulse width: 0.05 s; sampling width: 0.0167 s; pulse period: 0.5 s; quiet time: 2 s).

The differential pulse voltametric behavior of HepG2 cells (1.0 × 10^6^ cells/mL) on the composite-modified electrode MWCNT-COOH/α-Fe_2_O_3_/PGE was investigated (Figure 4). A weak oxidation peak was detected at +0.63 V on PGE, with a peak current of 1.29 µA (Figure 4A). After modification with MWCNT-COOH, the oxidation peak current at MWCNT-COOH/PGE increased to 2.18 µA, and the oxidation peak potential shifted to +0.60 V, attributed to the intrinsic excellent electron transfer capability and outstanding electrocatalytic performance of MWCNT-COOH (Figure 4B). Following the addition of α-Fe_2_O_3_, the oxidation peak current at MWCNT-COOH/α-Fe_2_O_3_/PGE further increased to 3.47 µA, with a slight decrease in oxidation peak potential by 0.005 V to +0.595 V compared to MWCNT-COOH/PGE (Figure 4C). This improvement is due to the good catalytic performance of α-Fe_2_O_3_, and the synergistic effect between α-Fe_2_O_3_ and MWCNT-COOH enhances the conductivity of α-Fe_2_O_3_. Figure 4D displays a comparative graph of the signals obtained from the cell lysate using the three types of electrodes after baseline correction, facilitating the observation of changes in the oxidation peak current across different electrodes.

**Figure 5:**
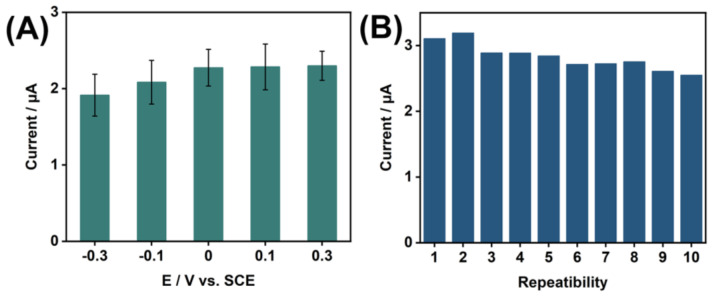
(**A**) Effect of enrichment potential on electrochemical signals of HepG2 cells detected by MWCNT-COOH/α-Fe_2_O_3_/PGE. (**B**) Repeatability of MWCNT-COOH/α-Fe_2_O_3_/PGE (Cell density: 1 × 10^6^ cells mL^−1^).

The influence of various enrichment potentials on the oxidation peak current of HepG2 cells was analyzed (Figure 5A). At negative potentials, the measured electrochemical signal intensity decreased. At an enrichment potential of 0 V or positive values, there was no significant effect on the electrochemical signal. Higher enrichment potentials may lead to increased background electrochemical reactions, generating background signals that interfere with the detection of the target analytes. Therefore, 0 V was chosen as the enrichment potential for detecting HepG2 cells using MWCNT-COOH/α-Fe_2_O_3_/PGE. The reproducibility of the electrode was assessed by measuring HepG2 cells ten consecutive times with the same MWCNT-COOH/α-Fe_2_O_3_/PGE (Figure 5B). The relative standard deviation (RSD) of the measured electrical signal was found to be 2.03%, indicating that the prepared MWCNT-COOH/α-Fe_2_O_3_/PGE exhibits good reproducibility.

### 3.4. Evaluation of the Toxicity of DBPs on HepG2 Cells

**Figure 6:**
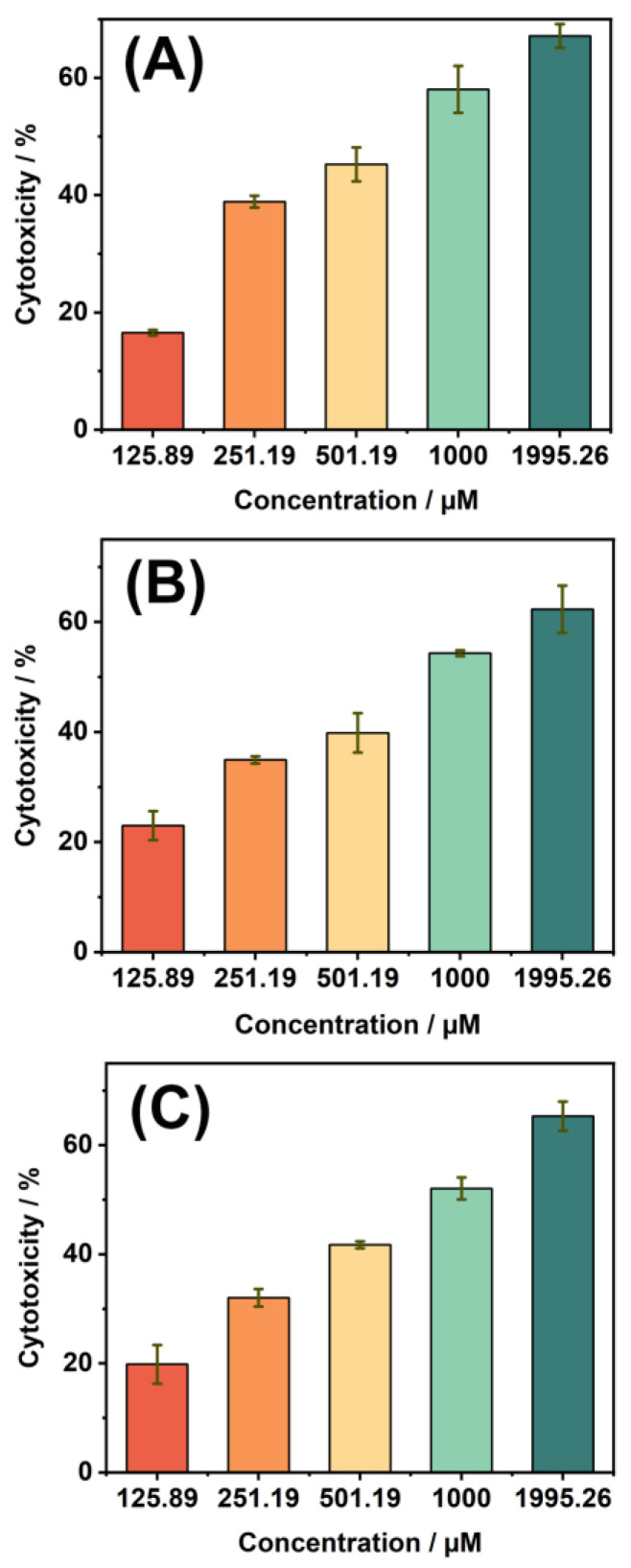
Cytotoxicity of 2-CPAN (**A**), 3-CPAN (**B**), and 4-CPAN (**C**) to HepG2 cells detected by the electrochemical sensor.

The constructed electrochemical sensor based on MWCNT-COOH/α-Fe_2_O_3_/PGE was utilized to study the cytotoxicity of three chlorophenyl acetonitrile DBPs (Figure S2 and Figure 6). Linear regression was performed on the logarithm of concentration versus cell toxicity to calculate the IC_50_ values for HepG2 cells (Table 1). All three DBPs exhibited cytotoxicity, with IC_50_ values of 831.76, 812.83, and 660.69 µM for 3-CPAN, 4-CPAN, and 2-CPAN, respectively. Studies on the cytotoxic mechanisms of various aromatic DBPs indicate that oxidative stress is one of the significant mechanisms leading to cytotoxicity [33]. The intracellular levels of reactive oxygen species (ROS) can partially reflect the extent of oxidative stress. The production of ROS, along with the imbalance in cellular redox state, can trigger excessive ROS production, resulting in destructive impacts on lipids, proteins, and DNA, ultimately leading to cell death [34]. Additionally, interactions between exogenous substances and the antioxidant enzyme system have also been confirmed as a mechanism of toxicity [35]. For example, exposure of cells to halogenated aromatic DBPs for a period results in a significant reduction in intracellular glutathione (GSH) levels [36]. Some DBPs can directly bind with GSH to form various glutathione conjugates, further reducing intracellular GSH levels [37]. GSH and the glutathione peroxidase enzyme system play crucial roles in the pathways of cellular redox homeostasis, and a decrease in GSH levels can weaken the cellular resistance to oxidative stress, increasing the risk of cell damage and death.

The order of cytotoxicity of chlorophenyl acetonitrile-type DBPs is 2-CPAN (ortho) > 4-CPAN (para) > 3-CPAN (meta). 2-CPAN (ortho) exhibits the strongest toxic effect on HepG2 cells, possibly due to the proximity of the chlorine atom to the cyano group, which affects the electronic density of the benzene ring and enhances the reactivity of the cyano group. This increased reactivity may lead to stronger interactions with biomacromolecules (such as proteins and DNA), thereby increasing cytotoxicity. The toxic effect of 4-CPAN (para) on HepG2 cells is higher than that of 3-CPAN (meta), which is consistent with the toxicity patterns of halogenated phenolic quinone-type DBPs in Chinese hamster ovary cells, where the cytotoxicity of 2,5-para-quinone is greater than that of 2,6-meta-quinone [38]. Research on the reactivity and genotoxicity of halophenolquinone isomers on DNA in human hepatocytes indicates that the dipole moment is a key structural parameter affecting DNA reactivity, cytotoxicity, and genotoxicity of HBQ isomers. The value of the dipole moment indicates the average distribution of charge and electrons within the molecule, and the reactivity of the molecule is strongly influenced by the dipole moment, with asymmetric molecules having a higher dipole moment than symmetric ones [39]. This may explain why the toxicity of 4-CPAN is greater than that of 3-CPAN.

**Figure 7:**
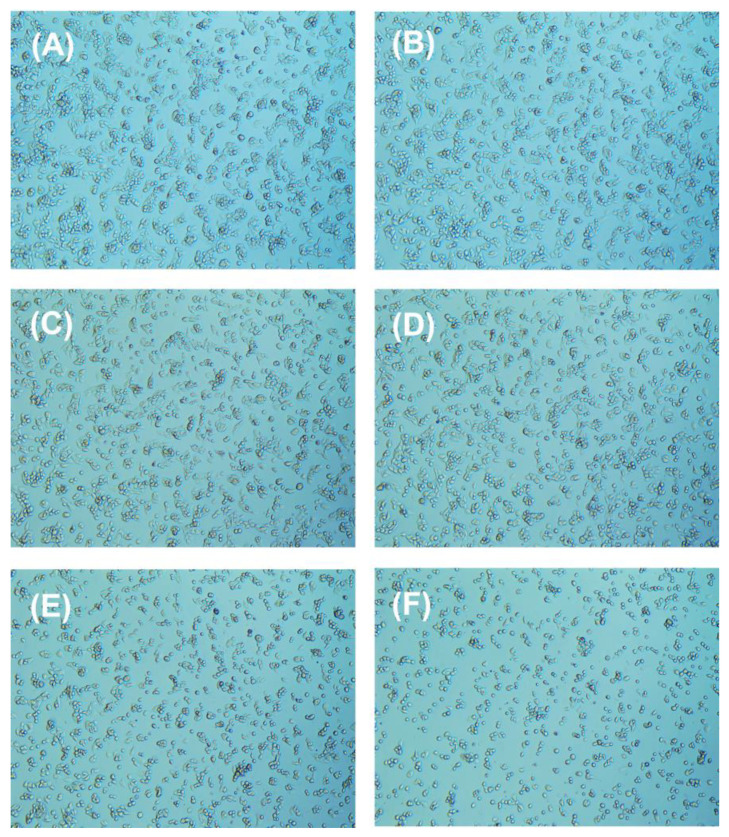
Cellular morphology of HepG2 cells (**A**) before and after treatment with (**B**) 125.89 µM, (**C**) 251.19 µM, (**D**) 501.19 µM, (**E**) 1000.00 µM, and (**F**) 1995.26 µM 2-CPAN for 36 h.

**Figure 8:**
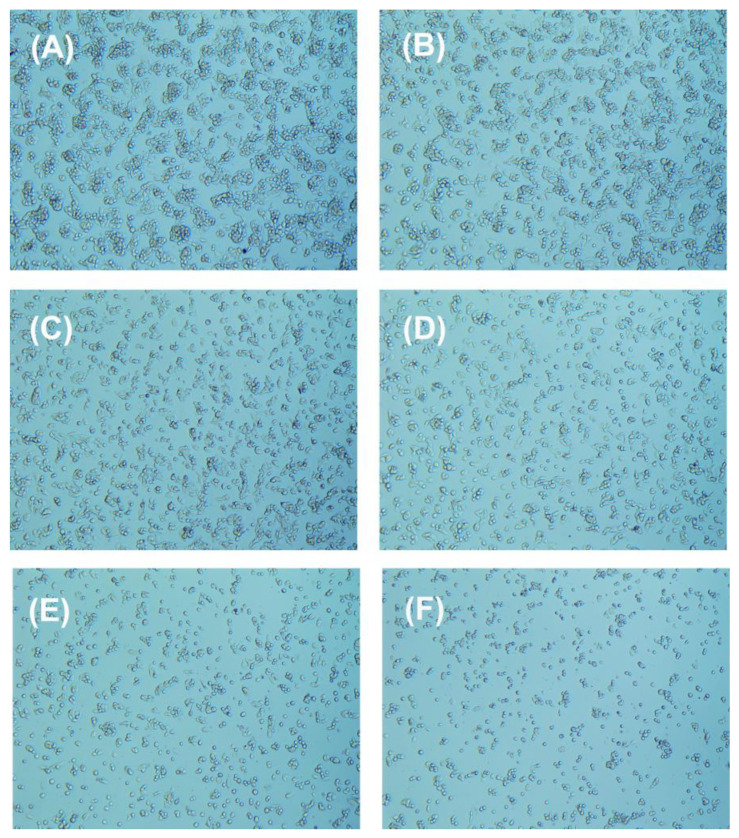
Cellular morphology of HepG2 cells (**A**) before and after treatment with (**B**) 125.89 µM, (**C**) 251.19 µM, (**D**) 501.19 µM, (**E**) 1000.00 µM, and (**F**) 1995.26 µM 3-CPAN for 36 h.

**Figure 9:**
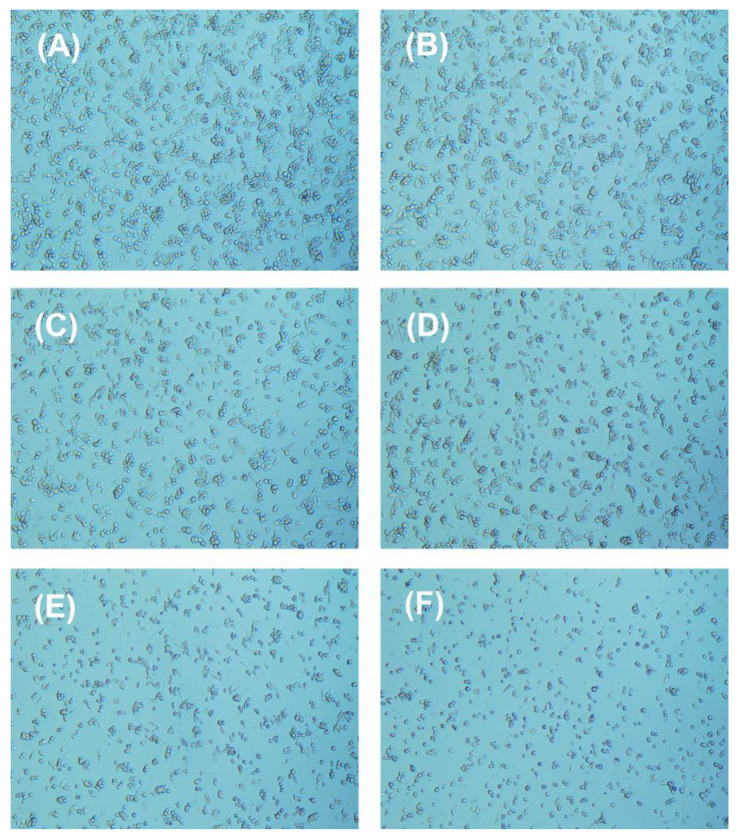
Cellular morphology of HepG2 cells (**A**) before and after treatment with (**B**) 125.89 µM, (**C**) 251.19 µM, (**D**) 501.19 µM, (**E**) 1000.00 µM, and (**F**) 1995.26 µM 4-CPAN for 36 h.

The changes in the morphology of HepG2 cells after exposure to chlorophenyl acetonitrile-type DBPs were studied (Figure 7, Figure 8 and Figure 9). With increasing concentrations of pollutants, the cell density gradually decreased. Most HepG2 cells in the control group displayed a spindle shape (Figure 7A, Figure 8A and Figure 9A), whereas the morphology of HepG2 cells after exposure showed significant changes, with cells becoming smaller and rounder, along with the appearance of numerous contracted dead cells (Figure 7B–F, Figure 8B–F and Figure 9B–F), indicating that all three chlorophenyl acetonitrile-type DBPs can inhibit the activity of HepG2 cells.

## 4. Conclusions

In this study, we developed an electrochemical cell sensor utilizing MWCNT-COOH/α-Fe_2_O_3_/PGE, showcasing excellent electrocatalytic performance. Compared to traditional cell toxicity detection methods, the electrochemical cell sensor has advantages such as simplicity, speed, and being label-free. Furthermore, the pencil graphite electrodes used in this study are only one-thousandth the cost of conventional glassy carbon electrodes, significantly reducing research costs. We investigated the toxicity of DBPs including 2-CPAN, 3-CPAN, and 4-CPAN based on changes in the electrochemical signals of HepG2 cells. Our findings indicate that 2-CPAN exhibits the highest level of toxicity to HepG2 cells, followed by 4-CPAN, while 3-CPAN demonstrates the lowest level of toxicity. Changes in cell morphology also reflect the varying toxicity levels associated with different types and concentrations of DBPs. This study provides technical support and scientific evidence for the detection of toxicity and the safety assessment of DBPs in water environments. In addition to cytotoxicity detection, the sensor can also be promoted and applied in other fields for detecting cellular nucleotide metabolic products, such as assessing cellular metabolic function and studying drug performance.

## Figures and Tables

**Table 1:** The linear equation between the concentration logarithm (*X*) of different DBPs and their cytotoxicity (*Y*) and their IC_50_ values to HepG2 cells.

Toxicant	Signal	IC_50_
2-CPAN	*Y* = 35.61 *X* − 55.43 (r = 0.976)	660.69 µM
3-CPAN	*Y* = 31.58 *X* − 39.88 (r = 0.962)	831.76 µM
4-CPAN	*Y* = 36.90 *X* − 61.61 (r = 0.916)	812.83 µM

## Data Availability

Data are contained within the article and Supplementary Materials.

## Supplementary Materials

The following supporting information can be downloaded at https://www.mdpi.com/article/10.3390/nano15020146/s1: Figure S1. Oxidation peak current diagram of 20 µM xanthine/guanine mixture at (A) MWCNT-COOH/PEG and (B) MWCNT-COOH/α-Fe_2_O_3_/PEG. Figure S2. The oxidation peak current values of cell lysates corresponding to different concentrations of (A) 2-CPAN, (B) 3-CPAN, and (C) 4-CPAN.
