# Supplementary material for: A Low-Cost Electrochemical Cell Sensor Based on MWCNT-COOH/α-Fe2O3 for Toxicity Detection of Drinking Water Disinfection Byproducts
Source: Nanomaterials (Basel). 2025 Jan 20;15(2):146. doi: 10.3390/nano15020146 (PMC11767749; doi:10.3390/nano15020146)
Supplement: Supplementary file 1 [file nanomaterials-15-00146-s001.zip › nanomaterials-3398351-supplementary.pdf]

Supporting information

A Low-Cost Electrochemical Cell Sensor Based on MWCNT-COOH/α-Fe_2O_3 for Toxicity Detection of Drinking Water Disinfection Byproducts

Ying Liu, Zhipeng Zhang, Yuling Wu, Huan Yang, Jiao Qu and Xiaolin Zhu**

School of Environment, Northeast Normal University, Changchun 130117, China
*Correspondence: zhuxl668@nenu.edu.cn; Tel: +86-431-8916-5600; Fax: +86-431-8916-5621

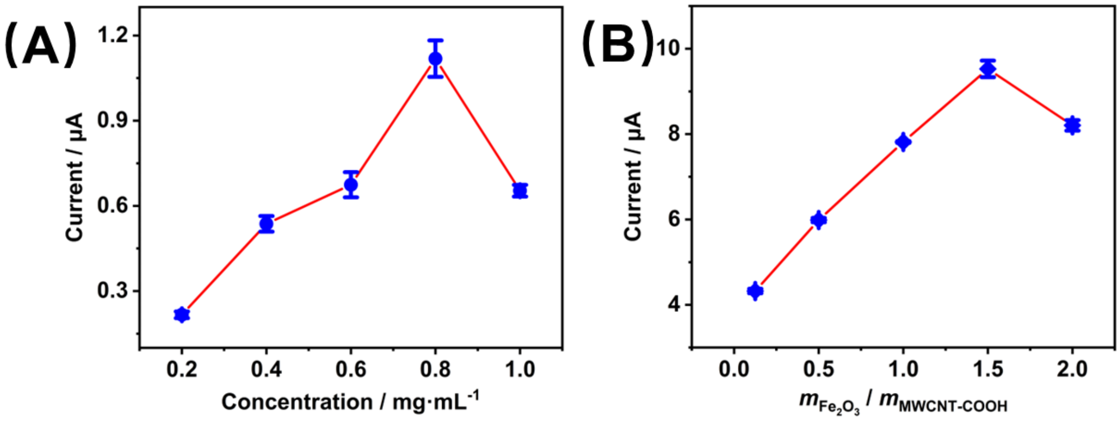

Figure S1 Oxidation peak current diagram of 20 μM xanthine/guanine mixture at (A) MWCNT-COOH/PEG and (B) MWCNT-COOH/α-Fe_2O_3/PEG.

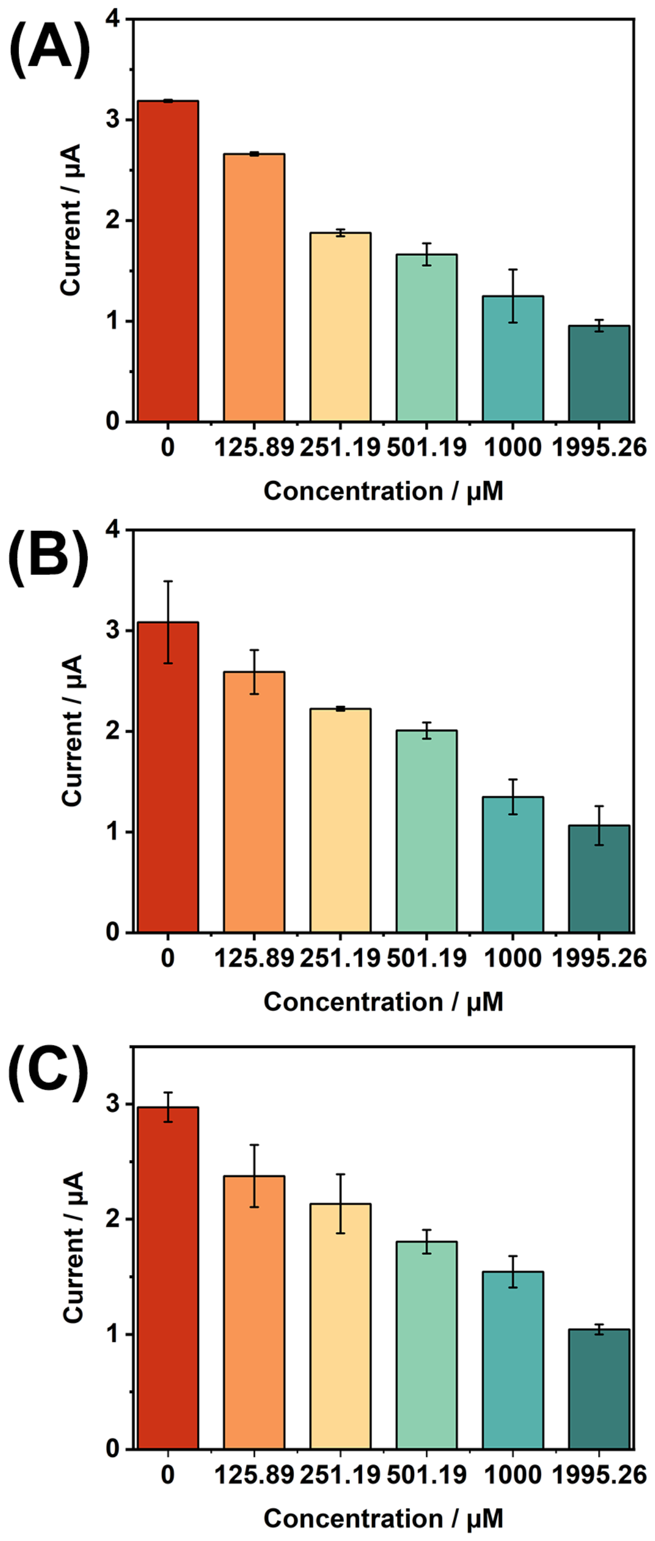

Figure S2 The oxidation peak current values of cell lysates corresponding to different concentrations of (A) 2-CPAN, (B) 3-CPAN, and (C) 4-CPAN.
